# Cancer in older patients: an analysis of elderly oncology

**DOI:** 10.3332/ecancer.2012.243

**Published:** 2012-02-02

**Authors:** V Swaminathan, RA Audisio

**Affiliations:** 1 Mersey Deanery, FY1 Southport DGH, Town Lane, Kew, Southport, PR8 6PN, UK; 2 Honorary Professor - University of Liverpool Consultant Surgical Oncologist - St Helens Teaching Hospital Marshalls Cross Road St Helens WA9 3DA, UK

## Abstract

Is it possible to define when someone is elderly? The worldwide population is growing not only in number but also in age; it is estimated that the population will increase to around 750 million by 2021. Two thirds of cancer occurs in the over 65 age groups. With an increasing elderly population, it can be derived that cancer will become a more prevalent condition. The burden of cancer on the medical profession will be even more apparent than before. In addition the elderly age group has different needs compared with younger oncology patients; there can be no ‘rule of thumb’ with the management of elderly illness. Factors such as frailty are significant when treating cancer in the older patients. The assessment of quality of life in older patients with cancer is also an important factor. Is it best for a patient to enjoy life as it is with cancer or aim for increased life expectancy by undertaking treatment with the threat of morbidity however severe during that period? The volume of scientific evidence currently available to support all the issues in geriatric oncology is greatly limited; almost all treatments designed for oncology are being tested in randomized clinical trials preferentially using younger cohorts of patients. Changes need to be made in order to further this field of medicine. Geriatric oncology is no longer a palliative field, as a healthy active life can now be expected by some older patients. The burden of oncology in the elderly will need to take a modern approach regarding the management of these patients.

## The ‘elderly’

This is a much-debated topic. Is it even possible to define when someone is elderly? People are able to perform at different levels within every age group; recently a British man, Fauja Singh, has completed a marathon at the age of 100 [1].

Currently the worldwide population is growing not only in number but also in age. The United States Census Bureau’s recent 2011 mid year world population calculations revealed that there are approximately 550 million people of the age 65 or over. This is almost 120 million more than 10 years prior in 2001. It is also estimated that this population will increase to around 750 million by 2021, a further 200 million (36%) over the next 10 years [2] (table 1 shows a breakdown of the population groups over 65 years of age).

Increasing numbers are also combined with increasing healthy life expectancy; 10 years at age 65 in most European countries (stretching beyond 15 years in some Scandinavian countries) [3]. With all this in mind, is it possible to define a category of elderly with such changing boundaries?; a chronological age of 70 is usually considered in the literature as a definition of ‘elderly’. The United Nations (UN) has no standard numerical criterion for ‘elderly’ at present, however the UN agreed cut-off is 60+ years of age when referring to the older population.

## Elderly oncology

As explained earlier, the number of elderly alive is increasing. With this increase in age, different diseases become more prevalent. It is known that two thirds of cancer occurs in the over 65 age groups. Thus, combined with an increasing elderly population, it can be derived that cancer will become a more prevalent condition. In addition, with advances in medical management, other conditions are affecting the population less, such as infective or vascular causes of illness. Thus, the burden of cancer on the medical profession will be even more apparent than before.

The burden of cancer in the population also has financial implications. For example, a course of Herceptin costs approximately £25,000 per annum on average [4], but furthermore cost effectiveness of the drug is quoted at £400,000 per recurrence prevented [5]. As the elderly population is increasing, more patients will develop cancer and require such treatments.

Is there gain to society from investing in such treatments for the elderly age groups? As explained, not only are the old getting older, but they are having increasing healthy life expectancy and active life years. Thus some of these individuals are very capable to provide valuable services to the community and for their vocation, above the age of 65, which was not seen in times gone by. In turn, if the elderly fall ill and require medical attention, attention should be paid, as it can be expected that they are ‘fit’ enough to recover and continue their ever growing active life expectancy. It is known that nearly a third of elderly individuals receive surgery during their last year of life [6], but there is no way to know for certain which patients will survive less than a year or not.

There can be no ‘rule of thumb’ with the management of elderly illness, as there is no longer a longer a clear cut-off/divide or standardisation for the health expectations of elderly age group.

## Additional needs of older patients with cancer

Furthermore to an increasing cost burden in older patients with cancer, the elderly age group has different needs compared with younger oncology patients. These patients may be less able to tolerate certain cancer treatments due to pharmacokinetics/dynamics; have other comorbidities; the presence of current polypharmacy even before the inclusion of any additional cancer therapy. Older patients may have functional problems, or difficulty with activities of daily living. Combined with a lack of access to transportation, social support or financial resources could result in reduced compliance, especially with more taxing and state-of-the-art modern management plans and treatments.

Malnutrition is a common problem in older patients with cancer. Malnutrition is both a cause and a consequence of ill health. It is common and increases a patient’s vulnerability to disease [7]. Patients may enter a state of high-refeeding risk, due to the nature of the illness. As a result a poor quality of life and poor adaptation to any stress events may occur. The National Institute for Health and Clinical Excellence (NICE) have set criteria to determine patients at high risk of refeeding, as shown in table 2.

In addition, poor nutritional status has implications regarding the success of any oncology treatment initiated. Adequate nutritional intake is the condition sine qua non which can make possible any attempt of aggressive oncologic therapies which are validated in adult subjects.[8].

## The concept of frailty

A close collaboration by geriatricians over the past years has allowed for better understanding of the interaction between geriatric syndromes and cancer management. The identification of frailty is recommended when treating cancer in the older patients. It is thought that this could be incorporated through a Comprehensive Geriatric Assessment (CGA). This is however time consuming. Quicker tools have thus been created (GFI, VES13, TUG) with the purpose of screening older patients with cancer for frailty, as described before, age only is not a sufficient marker to characterize these patients in modern society [9].

## Quality of life

This is an important concept; crucial to decisions made regarding the management of elderly patients, and can lead to the prolongation of one’s life, but often with consequences. There is much debate around quality of life in older patient with cancer. Is a patient best enjoy life as it is or aim for increased life expectancy with the threat of morbidity however severe during that period? Pancreatic cancer is an example of this dilemma. A patient may undergo an operation to help prolong life expectancy, but in turn undergoes major surgery off high risk and could end up with several comorbidities afterwards; no evidence exists to suggest post operative quality of life or wellbeing is increased after major surgery. The patient also spends much of their remain life in and out of the hospital pre and post operatively, which may reduce the patient’s quality of life for their remaining years.

## Making a decision

Historically a paternalistic approach was taken when deciding on future management of older oncology patient. Due to the nature of the illness, most patients will feel overwhelmed, thus putting their trust into the hands of the doctors. It is well known that surgery is less preferred as patient age increases, due to increasing comorbidities and reduced capacity to cope with the stress of such aggressive measures. For example, the risk of peri-operative death increases with age (overall mortality rate of 1.2% within 30 days of surgery for the general population, increased to 2.2% in 60–69 years old, 2.9% in 70–79 years old, 5.8–6.2% in those aged > 80 years and 8.4% in those aged > 90 years) [10–14]. Major surgery further increases this risk, leading to a 19.8% mortality rate in the latter group [15].

The decision to operate on a patient was thus based on whether the surgeon thought it was right or not. This method is very subjective, thus similar patients would receive different treatments and routes of management. This is also heavily influenced by a surgeon’s experience also; a younger or less experienced individual may prefer more modern, lower risk management strategies, chemotherapy or radiotherapy.

However, with identification of nutritional status, use of frailty screening tools, as well as nomograms that can help predict the need for certain operative procedures, the management of cancer in older patients can be more tailored. In addition, patient care should improve, as more appropriate management will be undertaking, using the available tools to guide the decision-making. This also helps remove an ageist approach to the management of older patients with cancer, as decisions can be made based on an individual-by-individual basis.

## Current evidence base

The volume of scientific evidence currently available to support these issues is greatly limited; almost all treatments designed for oncology are being tested in randomized clinical trials preferentially using younger cohorts of patients. It is thus an oncological priority to expand the use of older cohorts of patients in cancer research, due to the different challenges this cohort presents with regard to the management of cancer, and the growing medical need and numbers in the elderly oncology group. Geriatric oncology is no longer a palliative field, as healthy active life can now be expected by some older patients.

## Conclusion

The field of onco-geriatrics is vastly expanding. The demand from older patients is increasing, and is predicted to continue to expand for the foreseeable future. Life expectancy has increased, and in turn has patient expectations regarding the quality of their lives in the latter decades of age. The burden of oncology in the elderly will need to take a modern approach regarding the management of these patients. The use of screening and predictive tools can help make better decisions for these patients. Continued collaboration between organisations has also helped to develop the management of these patients; the International Society of Geriatric Oncology (SIOG) was founded in 2000 with a purpose to advance the art, science and practice of oncology in elderly patients and maintain a high common standard of healthcare in elderly patients with cancer [16]. This and other similar steps forward will hopefully bring a more tailored and higher standard of care to older oncology patients.

## Figures and Tables

**Table 1:**
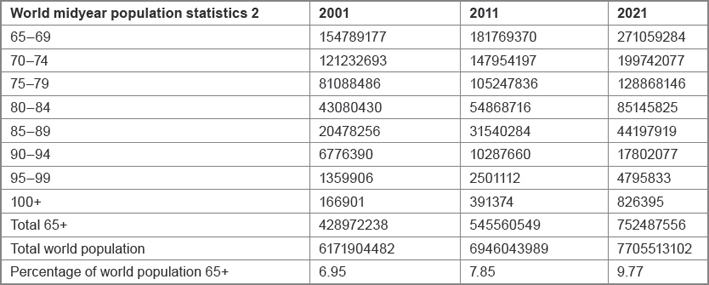
Breakdown of the population groups over 65 years of age

**Table 2:**
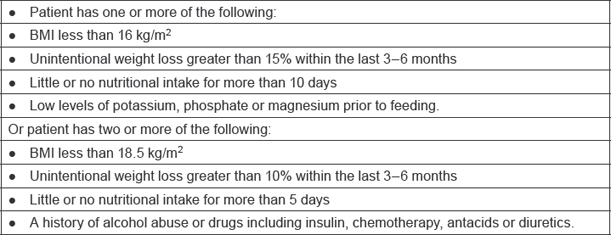
Criteria to determine patients at high risk of refeeding [7]
